# Quantitative micro-elastography: imaging of tissue elasticity using compression optical coherence elastography

**DOI:** 10.1038/srep15538

**Published:** 2015-10-27

**Authors:** Kelsey M. Kennedy, Lixin Chin, Robert A. McLaughlin, Bruce Latham, Christobel M. Saunders, David D. Sampson, Brendan F. Kennedy

**Affiliations:** 1 Optical+Biomedical Engineering Laboratory, School of Electrical, Electronic & Computer Engineering, The University of Western Australia, 35 Stirling Highway, Crawley WA 6009, Australia; 2 PathWest, Fiona Stanley Hospital, Robin Warren Drive, Murdoch, WA 6150, Australia; 3 School of Surgery, The University of Western Australia, 35 Stirling Highway, Crawley, WA 6009, Australia; 4 Breast Clinic, Royal Perth Hospital, 197 Wellington Street, Perth, WA 6000, Australia; 5 Centre for Microscopy, Characterisation & Analysis, The University of Western Australia, 35 Stirling Highway, Crawley, WA 6009, Australia

## Abstract

Probing the mechanical properties of tissue on the microscale could aid in the identification of diseased tissues that are inadequately detected using palpation or current clinical imaging modalities, with potential to guide medical procedures such as the excision of breast tumours. Compression optical coherence elastography (OCE) maps tissue strain with microscale spatial resolution and can delineate microstructural features within breast tissues. However, without a measure of the locally applied stress, strain provides only a qualitative indication of mechanical properties. To overcome this limitation, we present quantitative micro-elastography, which combines compression OCE with a compliant stress sensor to image tissue elasticity. The sensor consists of a layer of translucent silicone with well-characterized stress-strain behaviour. The measured strain in the sensor is used to estimate the two-dimensional stress distribution applied to the sample surface. Elasticity is determined by dividing the stress by the strain in the sample. We show that quantification of elasticity can improve the ability of compression OCE to distinguish between tissues, thereby extending the potential for inter-sample comparison and longitudinal studies of tissue elasticity. We validate the technique using tissue-mimicking phantoms and demonstrate the ability to map elasticity of freshly excised malignant and benign human breast tissues.

The mechanical properties, structure, and function of tissues are linked and altered by disease^1^. Clinicians palpate tissues to detect changes in stiffness indicative of disease; for example, breast tumours are typically discovered as palpable lesions. Palpation provides a subjective, qualitative measure of mechanical properties, but in recent decades, elastography techniques have been developed to quantitatively image these properties^2^. Elastography uses medical imaging to track the deformation of tissue under an applied load and relates the deformation to a mechanical property or parameter, typically stiffness, or elasticity, which is mapped onto an image. Elastography was first developed based on ultrasound^3^ and later on magnetic resonance (MR) imaging^4^, and these modalities are now in clinical use for applications including monitoring of liver disease^5^ and diagnosis of breast lesions^6^. The spatial resolution of these techniques ranges from 100s of micrometres^7^ to several millimetres^8^, allowing visualization of macroscale tissue features. However, disease-related mechanical changes also manifest on much finer scales. For example, in breast cancer, studies using atomic force microscopy have shown that tumour cells are softer than normal cells^9^, which allows them to more easily migrate through tissue, and, at the edge of an infiltrating tumour, tumour cells are intermingled with adipose and fibrous tissues of the breast, creating a mechanically heterogeneous microenvironment, the details of which cannot be resolved using the clinically available elastography techniques.

The ability to detect microstructural features of breast tumours could help to guide breast-conserving surgery, in which the goal is to completely remove malignant tissue whilst sparing normal breast tissue. Tumour cells found at or near the boundary (margin) of the excised tissue by post-operative histopathology are associated with an increased risk of local recurrence, and, currently, ~20–60% of patients must undergo multiple surgeries to remove residual tumour^10^,^11^. Surgeons largely rely on palpation to guide excision or, in the case of non-palpable lesions, use preoperative imaging to find the tumour site and estimate its size; however, these techniques cannot precisely delineate tumour boundaries during surgery. Intraoperative histological assessment, such as frozen section histology and imprint cytology, may also be used to test margins for the presence of tumour, but these are time-consuming (~25 minutes on average) and have not led to a significant reduction in re-excision rates^12^,^13^. Likewise, intraoperative specimen radiography, used mainly in cases of *in situ* cancers, has not been shown to reduce re-excision rates^12^. Intraoperative ultrasound guidance of excision has been shown to reduce re-excision rates for invasive cancers^14^,^15^, but ultrasound typically cannot visualize *in situ* or multifocal cancers^16^,^17^. Thus, new intraoperative guidance and/or margin assessment techniques are expected to improve patient outcomes in breast-conserving surgery. Imaging the mechanical properties of breast tissue on the microscale could potentially contribute to a solution by highlighting areas of malignancy otherwise missed by palpation or other clinical modalities.

Using optics to image the mechanical properties of tissue, *i.e.*, optical elastography, has been developed using modalities including optical coherence tomography (OCT)^18^,^19^,^20^, laser speckle imaging^21^, and Brillouin microscopy^22^. Optical coherence elastography (OCE), based on OCT, can offer spatial resolutions in the range 1–100 μm and possesses several advantageous features for imaging in a clinical setting, including high-speed, 3D imaging (volumes have been acquired in ~5 seconds^23^) and *in vivo* capabilities^24^,^25^, as well as the potential for delivery via endoscopic^26^,^27^ and needle-based probes^28^,^29^. OCT is also exquisitely sensitive to tissue motion; the change in phase of the complex OCT signal is proportional to axial sample displacements as small as 10s of picometres^30^,^31^. This sensitivity allows detection of smaller changes in mechanical properties than is achievable using clinical ultrasound elastography or MR elastography, which are sensitive to displacements of 100s of nanometres to micrometres^32^,^33^.

We have previously developed a compression OCE technique that maps axial strain to form 3D images, referred to as micro-elastrograms^34^,^35^, which show mechanical variations within human breast tissues^36^. We have shown that strain can distinguish normal fibrous tissue (stroma) from regions of invasive malignancy, as well as delineate features, such as ducts and vessels, often with additional contrast to that observed in OCT^34^,^36^. Although strain provides contrast within an image, it is an incomplete indication of mechanical properties. Strain is inversely proportional to elasticity only under the assumption of uniform stress throughout the sample. Without knowledge of the local stress, strain only qualitatively represents mechanical properties, limiting image contrast in some scenarios and making it challenging to compare results across time points or across samples. In breast tissue, the qualitative nature of strain imaging can limit the ability to distinguish tissues types, including regions of solid tumour and solid stroma; although their elasticity values are likely to differ, both produce homogeneous strain. A measure of stress combined with strain would allow quantification of elasticity, which may enhance the ability to reliably identify malignant breast tissues and would facilitate inter-sample comparison.

We recently demonstrated OCT-based tactile imaging to map the stress applied to the surface of tissue under compression. This technique, termed optical palpation^24^,^37^, employs a compliant, translucent silicone sensor placed between the sample and the compression plate. OCT is used to measure the strain of the sensor, and the stress-strain behaviour of the sensor material is well characterized, such that sensor strain may be mapped to stress. The result is a 2D map of the stress applied to the sample surface.

Here, we combine compression OCE and optical palpation to simultaneously perform strain and stress imaging and produce images of tissue elasticity: quantitative micro-elastograms. This is the first quasi-static, compression OCE technique to quantify elasticity, rather than rely on contrast based on strain alone. OCE techniques based on shear wave imaging can also directly estimate elasticity from the wave velocity^38^,^39^. However, these techniques assume mechanical homogeneity of the sample over the shear wavelength and have not shown the ability to resolve microstructural features such as those visible in compression OCE images^34^,^35^,^36^. In addition, quasi-static compression OCE is more suited to rapid, wide-field (several cm) scanning of tissue desired for many surgical applications^19^,^23^. We utilize our technique to present the first quantitative maps of breast elasticity on the micro- to milliscale, with potential for intraoperative deployment. Previous studies in breast tissues, at the nano- to microscale using atomic force microscopy^9^ and at the macroscale using ultrasound elastography^6^ and bulk indentation^40^, have shown that the elasticity of breast tumours is distinct from that of normal breast tissue. Here, we enable quantitative imaging of elasticity at the intermediate scale of tissue microstructure, a scale critical in the detection of tumour boundaries in breast-conserving surgery.

In this paper, we describe our approach to quantitative micro-elastography and validate its accuracy through 3D quantitative micro-elastograms of tissue-mimicking phantoms with elasticity in the range 5–180 kPa. Results in fresh, excised human breast tissues show the ability to distinguish tissue types based on elasticity, with one example exhibiting more than 100 times higher contrast between tumour and stroma compared to strain imaging, and indicate the potential of quantitative micro-elastography to aid in intraoperative guidance of breast cancer surgery.

## Working Principle and Results

### Quantitative micro-elastography

**Figure 1:**
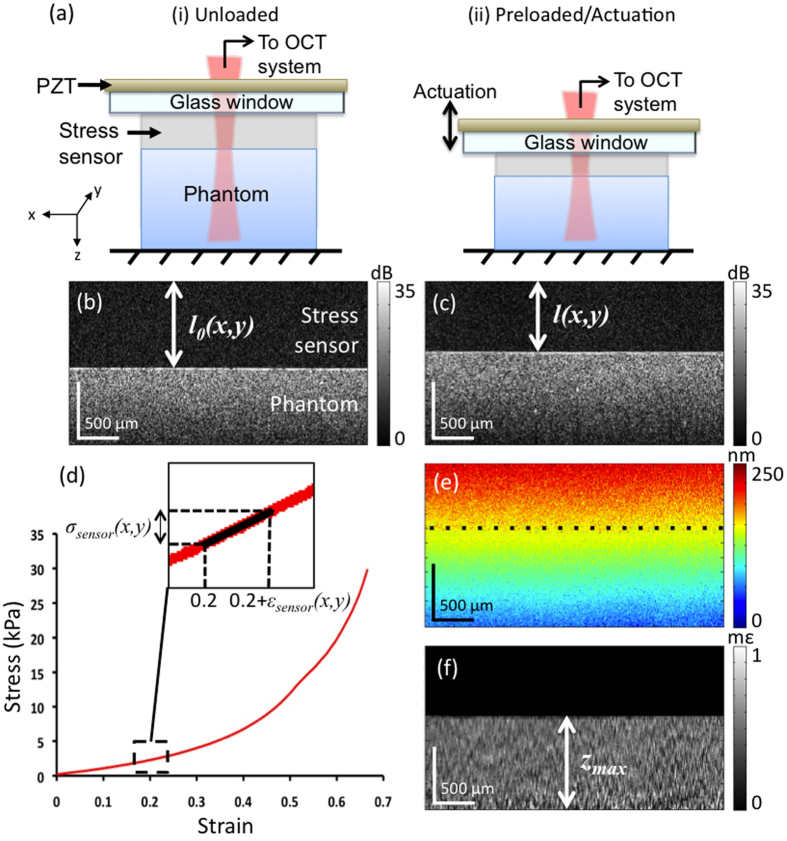
Quantitative micro-elastography working principle. (**a**) Schematics of the (i) unloaded and (ii) preloaded setup. (**b**,**c**) Corresponding OCT B-scans indicating the original and preloaded sensor thicknesses, 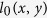 and 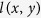, respectively. (**d**) Stress-strain curve of the sensor material. In the inset, the stress, 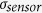, due to actuation is estimated using the tangent to the curve at the preload strain (0.2 in this example). (**e**) B-scan view of the displacement due to micrometre-scale actuation; dotted line indicates the sensor-sample interface. (**f**) Corresponding strain in the sample, indicating the depth range over which strain is averaged, 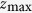, to generate a 2D quantitative micro-elastogram.

Figure 1 illustrates the working principle of quantitative micro-elastography. A translucent, silicone sensor is placed between a glass window, through which imaging is performed, and the sample. A preload is applied by bringing a rigid plate into contact with the opposite surface. The preload applied to each sample is the minimum required to ensure uniform contact between the sensor and sample, corresponding to a strain in the range 0.05–0.2, depending on the amount of sample thickness unevenness. Illustrations of the unloaded and preloaded setup are shown in (i) and (ii) in Fig. 1(a), and corresponding 2D OCT images (B-scans) are shown in Fig. 1(b,c). The sensor presents a low level of intrinsic optical scattering. The test sample is a homogeneous silicone phantom with stiffness ~5 times that of the sensor and with titanium dioxide scatterers incorporated (2 mg/mL) to provide optical backscattering. The initial and preloaded sensor thicknesses, 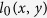 and 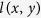, respectively, are measured at each lateral position in a B-scan using an edge detector based on the Canny filter to locate the sensor-sample interface. The change in thickness is used to calculate the axial sensor strain at each lateral position. We note that because this is a ratio of two thicknesses, it is not necessary to know the refractive index of the sensor material. The corresponding axial stress values are estimated from the stress-strain curve of the material, Fig. 1(d). These stress values correspond to those used to generate optical palpation images in our previous work^24^,^37^.

In the preloaded state, an annular, piezoelectric actuator (PZT in Fig. 1(a)) that is fixed to the window introduces step-wise, micrometre-scale axial displacement (amplitude up to 2.2 μm) to the sensor surface, with a frequency in the range 2–25 Hz. The actuation is synchronized with imaging such that sequential B-scans are acquired in two different loading states^34^. As the increment in load occurs between the acquisition of B-scans, the sensor and sample are stationary during data acquisition. The depth-resolved axial displacements in the sensor and the sample, as shown in Fig. 1(e), are calculated from the phase difference between corresponding OCT axial scans in sequential B-scans. The depth-resolved axial strain in the sample, as shown in Fig. 1(f), is estimated using weighted-least-squares regression to the displacement with respect to depth^41^ and incorporating phase unwrapping, as detailed previously^34^. We do not calculate depth-resolved strain in the sensor, as it is homogeneous with depth; thus, this region is masked out in Fig. 1(f). Furthermore, to do so would require greater scattering signal in the sensor, as accurate, phase-sensitive strain estimation relies on high OCT signal-to-noise ratio (SNR)^41^,^42^, and this would degrade image quality in the underlying sample. Rather, local strain in the sensor is calculated using the measured displacement at the sensor-sample interface, *d*_*l*(*x*,*y*)_, (along the dotted line in Fig. 1(e)), where the OCT SNR is generally high due to a local change in refractive index. As OCT is performed here in a common-path configuration (see Methods), using the window-sensor interface as the reference, displacements are measured relative to this interface. Thus, *d*_*l*(*x*,*y*)_ represents the change in sensor thickness due to the micrometre-scale actuation and is divided by *l*(*x, y*) to get local strain in the sensor, 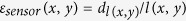. This sensor strain can then be used to estimate the corresponding stress, 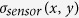, assuming that the sensor material behaves according to the slope of the tangent to the stress-strain curve, 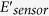, about the point of preload strain (indicated in the inset in Fig. 1(d)):


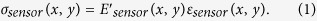


This stress, typically in the range 1–500 Pascals (Pa), is divided by the local strain in the sample to calculate local elasticity, 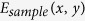, which is mapped into a 2D micro-elastogram in the *xy*-plane:


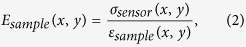


where *ε*_*sample*_(*x, y*) is calculated by averaging strain over the depth range *z*_max_, indicated in Fig. 1(f). The latter is chosen based on the sample’s optical attenuation and is set to a fixed distance beyond the sensor-sample interface (0.8 mm in this example). Assuming constant stress with depth, 3D micro-elastograms can also be constructed by dividing the stress at the surface by the depth-resolved strain in the sample. In general, *E*_*sample*_ represents the elasticity of the sample at the applied preload. In the linear elastic regime, *E*_*sample*_ represents the Young’s modulus of the sample.

**Figure 2:**
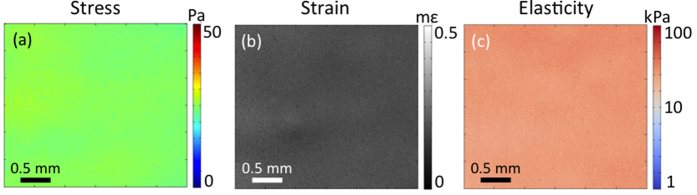
Stress and strain are combined to form a quantitative micro-elastogram of a homogeneous phantom. (**a**) *En face* map of stress measured by the sensor; (**b**) Corresponding strain in the sample; (**c**) Quantitative micro-elastogram generated by dividing (**a**) by (**b**).

Figure 2(a–c) show 2D, *en face* maps of local stress, local strain, and the resulting quantitative micro-elastogram, respectively, for the test sample in Fig. 1. Stress and strain are plotted on a linear scale. Due to the large range of elasticity measured in tissue, elasticity is plotted on a logarithmic scale. As expected for this homogeneous sample, the images in Fig. 2 are relatively uniform, with <10% standard deviation (27.0 ± 2.6 kPa) in elasticity over the field of view. The variation is likely due to sample imperfections arising from the fabrication process, rather than due to system noise (see Supplementary Note for a discussion of system sensitivity, independent of sample heterogeneity).

### Validation of elasticity measurements

**Figure 3:**
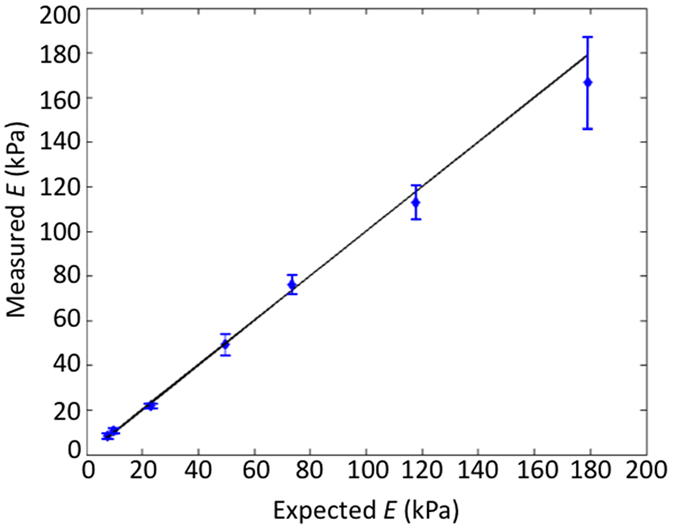
Validation of elasticity measurements of homogeneous silicone phantoms using quantitative micro-elastography (measured) against standard compression tests (expected).

To assess the accuracy with which elasticity is quantified using this technique, we performed measurements on seven homogeneous silicone phantoms with expected elasticity in the range 5–180 kPa, as measured using standard compression tests (expected elasticity varied by <10% for strains <0.2; see Methods). These elasticity values correspond to reported ranges for tissues, including benign and malignant breast tissues under compression^40^,^43^,^44^. 2D quantitative micro-elastograms were generated using *z*_max_ = 0.6 mm. Figure 3 plots the expected elasticity values versus those measured using quantitative micro-elastography. The solid line represents an ideal system (expected = measured), the diamonds represent the mean measured elasticity for a 6 × 6 mm (*x* × *y*) region across four independent measurements of each sample (indicating accuracy), and the error bars represent the mean error across the four measurements (indicating repeatability). In each case, the measured elasticity matched to within 8% of the expected elasticity, and inter-measurement variability was <12%. Repeatability degraded slightly with higher elasticity, which is attributed to the resistance of stiffer samples to conform evenly to the compression plates, resulting in inconsistent boundary conditions across measurements.

### 3D quantitative micro-elastography of structured phantoms

The stress-sensing method used here maps stress applied to the sample surface. In general, depth-resolved measurements of stress are needed to accurately evaluate sample elasticity in 3D; however, for certain sample geometries, the assumption of uniform stress with depth is sufficiently valid to allow accurate, depth-resolved estimates of elasticity using only the surface stress and depth-resolved strain. We previously used finite element analysis to demonstrate two cases in which stress is uniform or approximately uniform with depth^45^: 1) in layered structures, in which stress is theoretically uniform in the absence of friction; and 2) in samples containing stiff inclusions. The latter produces mostly uniform stress fields, except for stress concentrations at inclusion boundaries, which intensify with greater elasticity contrast between the inclusion and matrix. Here, we demonstrate the ability of quantitative micro-elastography to accurately map depth-resolved elasticity in these cases.

**Figure 4:**
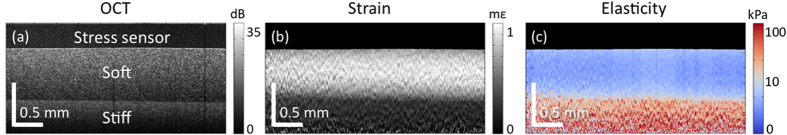
Depth-resolved quantitative micro-elastography of a bilayer phantom . (**a**) OCT B-scan after preload, (**b**) strain in the sample due to micrometre-scale actuation, and (**c**) quantitative micro-elastogram.

Figure 4 shows depth-resolved quantitative micro-elastography results in a bilayer phantom consisting of a 600-μm soft layer cured on top of a 500-μm stiff layer, with expected elasticity values of 21 kPa and 90 kPa, respectively (estimated by calculating strain in each layer due to preload and finding the tangent to the materials’ independently measured stress-strain curves at these strain values). Figures 4(a–c) show B-scan views of OCT, strain, and elasticity, respectively. The images are cropped in depth where the mean OCT SNR was <3 dB to avoid inaccurate displacement estimates. Figure 4(b) shows that the softer layer undergoes greater deformation than the stiffer layer, as expected. The mean elasticity values, measured over 0.5 × 0.5 × 0.2 mm (*x* × *y* × *z*) regions, are 24 kPa and 80 kPa for the soft and stiff layers, respectively, matching the expected values to within 15%. Discrepancies may be due to uncertainty in the preload applied to each layer; as the phantom materials have nonlinear stress-strain behaviour, their effective elasticity values depend on the amount of preload.

**Figure 5:**
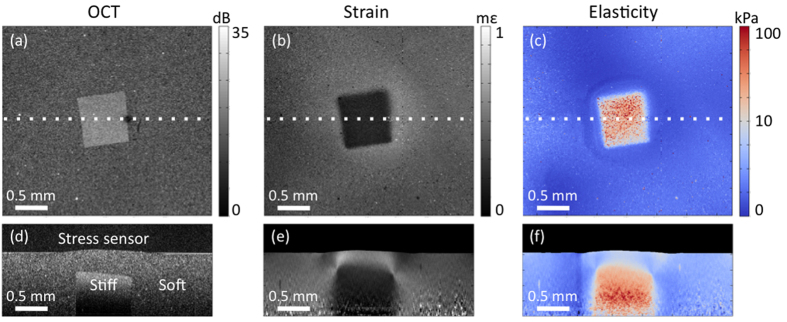
3D quantitative micro-elastography of an inclusion phantom. *En face* (top) and B-scan views (bottom, in plane indicated by dotted lines) of (**a**,**d**) OCT, (**b**,**e**) strain, and (**c**,**f**) elasticity.

Figure 5 shows 3D results in an inclusion phantom consisting of a ~750 μm cubic inclusion embedded ~500 μm below the surface in a silicone matrix with total thickness 1.5 mm. The expected elasticity values of the inclusion and matrix, calculated using the same procedure as described for the bilayer phantom, are 28 kPa and 5.5 kPa, respectively. Figures 5(a–c) show *en face* views of OCT, strain, and elasticity, and Fig. 5(d–f) show B-scan views (cropped in depth where the mean OCT SNR was <3 dB) in the plane indicated by the dotted lines in Fig. 5(a–c). Mean elasticity measured over 0.5 × 0.5 × 0.2 mm (*x* × *y* × *z*) regions are 25 kPa and 5.1 kPa for the inclusion and the matrix, respectively, matching the expected values to within ~10%. In Fig. 5(f), elevated elasticity is measured in the matrix above the inclusion, corresponding to points of expected stress concentration^45^. We note that the increase in elasticity measured here may also be genuine, as the matrix material exhibits nonlinear stress-strain behaviour and, thus, is expected to have higher effective elasticity in regions under higher strain/stress.

### Quantitative micro-elastography of human breast tissues

To demonstrate quantitative micro-elastography of breast tissue, we imaged freshly excised tissues from patients undergoing surgical removal of breast tumours. For these samples, we generated 2D, *en face* quantitative micro-elastograms, rather than 3D, as the uniformity of stress with depth in these tissues is unknown. Strain was averaged over a depth range of 300 μm beyond the sensor-sample interface, as the OCT SNR below this depth was generally too low for accurate strain estimation; thus, the resulting quantitative micro-elastograms represent the average sample elasticity over this depth range. We overlaid each quantitative micro-elastogram on a single, *en face* OCT image, sectioned 50 μm below the sensor-sample interface, to provide structural context for the elasticity values. We masked out regions of adipose in the quantitative micro-elastograms, as OCT easily distinguishes adipose based on its honeycomb structure, and the strain estimation algorithm performs sub-optimally in adipose due to large fluctuations in the underlying OCT SNR^34^,^36^. Negative values were also excluded from the elasticity data (see Discussion for a description of the origin of these negative values).

**Figure 6:**
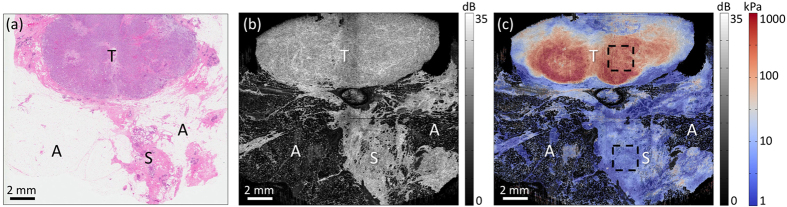
Quantitative micro-elastography of a malignant breast tumour. (**a**) H&E histology. (**b**) *En face* OCT image. (**c**) Fused *en face* OCT and quantitative micro-elastogram. Elasticity is plotted on a logarithmic scale. Dashed boxes indicate regions over which mean elasticity values were calculated. A = adipose, S = stroma, T = tumour.

Figure 6 shows results in a tumour excised from a 60-year-old female undergoing mastectomy and classified as invasive ductal carcinoma. The sample contained a palpable, stiff region surrounded by what appeared, from macroscopic inspection, to be mainly adipose tissue. To bring the sensor into uniform contact with the tissue surface, a preload corresponding to ~0.2 bulk strain was applied. Figures 6(a–c) show haematoxylin and eosin (H&E) histology, the *en face* OCT image, and the quantitative micro-elastogram overlaid on OCT, respectively. These 1.95 × 1.95-cm images were constructed by stitching together four 1 × 1 cm images acquired with 0.5 mm of overlap^36^. Histology reveals a large, oval-shaped region of invasive tumour cells (T, stained purple), surrounded by a band of fibrous stroma (primarily collagen, stained pink). A region of primarily stromal tissue (S) extends into the adipose (A) and comprises a mixture of collagen, normal ductal structures, and microcystic structures. The quantitative micro-elastogram reveals that the region of tumour is two orders of magnitude stiffer than the normal region below and provides much greater contrast between the two regions than OCT alone. The mean elasticity values over 2 × 2 mm regions within the upper and lower dashed boxes in Fig. 6(c) are 410 kPa and 5 kPa, respectively. These values are in the range previously reported for invasive ductal carcinoma and normal fibroglandular tissues of the breast under compression (measured using indenters with diameter 4–5 mm)^43^,^44^.

**Figure 7:**
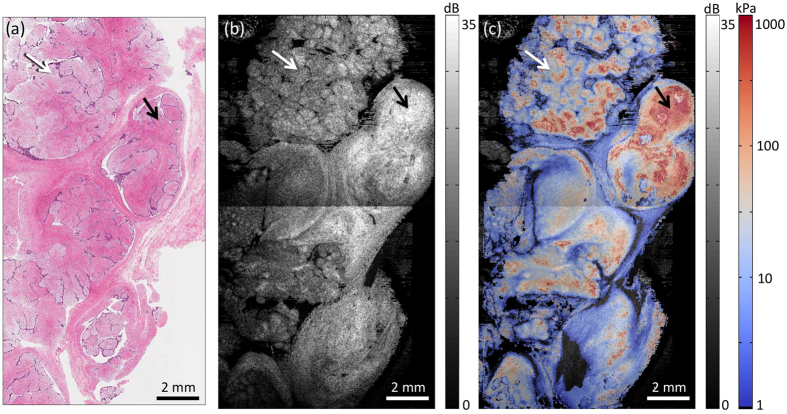
Quantitative micro-elastography of a fibroadenoma. (**a**) H&E histology. (**b**) *En face* OCT image. (**c**) Fused *en face* OCT and quantitative micro-elastogram. Elasticity is plotted on a logarithmic scale. Black arrows indicate a region of highly dense fibrous tissue. White arrows indicate a region comprised of small fibrous nodules delineated by epithelial cells.

Figures 7(a–c) show H&E histology, the *en face* OCT image, and the quantitative micro-elastogram overlaid on OCT, respectively, for a fibroadenoma removed from a 25-year-old female. As seen in the histology, the sample is comprised primarily of nodules of fibrous tissue, with small, slit-like regions of epithelial cells (stained purple) throughout. Due to its nodular structure, the sample had irregular thickness over the imaging field of view, and a preload corresponding to ~0.2 bulk strain was required to bring the sensor into uniform contact with the tissue surface. The change in intensity seen along a horizontal line in Fig. 7(b,c) is where two 1 × 1 cm images were stitched together with 0.5 mm overlap. Consistent with previous OCT imaging of fibroadenoma^46^, a region of denser fibrous tissue (stained darker pink), indicated by black arrows, corresponds to higher backscattering in the OCT image. In the micro-elastogram, this region has elevated elasticity compared to regions of less dense stroma. In a region comprised of small nodules separated by epithelial tissues, indicated by white arrows, the micro-elastogram reveals greater elasticity in the fibrous centre of the nodules, and lower elasticity at the cellular boundaries. This further demonstrates the ability to quantify the elasticity of microscale tissue features. Overall, the measured elasticity in this sample is in the range 15–450 kPa. The variation is likely due to varying density of the fibrous tissue, but could also partly be due to spatially varying preload, as fibroadenomas have been shown to be nonlinear in elasticity. For example, Wellman *et al.* reported elasticities of 45 kPa at 0.01 strain and 890 kPa at 0.2 strain^44^.

## Discussion

The results presented here constitute the first quantitative images of tissue elasticity acquired using quasi-static compression OCE. The novel combination of stress imaging using optical palpation with strain imaging using compression OCE enabled quantitative elasticity imaging of human breast tissues, with good correspondence to histopathology and OCT, and with values similar to those reported in the literature. We have previously reported on the use of strain imaging to provide mechanical contrast between breast tissues within an image, complementing the structural information in OCT^34^,^36^. Quantitative imaging further improves the ability to distinguish tissue types, especially those that have disparate stiffness but have similar structural characteristics, or present similar levels of strain. For example, in the case of the malignant breast tumour presented in Fig. 6, the mean strain values within the upper and lower dashed boxes in Fig. 6(c) are 0.27 mε and 0.35 mε, respectively, and the corresponding elasticity values are 410 kPa and 5 kPa. This represents a >100-fold increase in contrast between these tissues by quantifying elasticity. The additional contrast provided by quantitative micro-elastography is illustrated in Supplementary Figure S1, which shows the strain image corresponding to the results shown in Fig. 6 alongside the elasticity image presented in Fig. 6(c).

One of the next steps for quantitative micro-elastography is to perform validation studies to aid in the interpretation of tissue elasticity maps. This could be achieved by performing comparative studies with AFM. In addition, using picrosirius red staining, rather than H&E, could enable a better comparison between elasticity and collagen content.

A dynamic compression OCE technique based on kHz vibrational loading has also been proposed in which the sample is embedded in a medium of known elasticity, and the relative strain between the medium and sample is used to estimate sample elasticity^47^,^48^. However, this technique does not measure local stress and instead assumes that the medium used, agar, has constant elasticity^38^ (*i.e.*, is linear elastic), which has been shown to be accurate for strains <0.1^49^. Here, as local stress is quantified, we account for nonlinear behaviour of the sensor, allowing sample elasticity to be evaluated for a larger range of strains. Also, the dynamic technique relied on scattering signal in the agar to estimate strain^47^; here, we use a translucent sensor, which allows light penetration to the underlying sample to preserve image quality and OCE accuracy.

The axial resolution of quantitative micro-elastography is determined by the axial resolution of the strain measurement, *i.e.*, the depth range over which linear regression to displacement is performed (100 μm used here). To generate 2D *en face* micro-elastograms, such as those in Figs 6 and 7, strain is averaged over a depth range (300 μm in these samples) determined by the depth at which the OCT signal becomes too low for accurate displacement measurement. Thus, these images represent an average mechanical response of the samples over this depth range, resulting in some blurring of features compared to OCT *en face* images, which represent single, ~8-μm sections of the optical backscattering response of the sample. Extension to 3D imaging, as in Figs 4 and 5, allows accurate estimation of the depth-resolved elasticity where the assumption of constant stress with depth is sufficiently valid^45^. This assumption is likely to be valid in laminar tissues, such as skin^50^ or the epithelial tissues of the airway^51^. In more geometrically complex tissues, such as the breast tissues imaged here, stress is expected to vary with depth. However, the surface stress and depth-resolved strain measurements in this technique, as well as the structural information afforded by OCT, could serve as inputs to inverse methods that may be able to solve more accurately for the 3D distribution of elasticity^52^,^53^.

Assuming uniaxial compression of the sensor, the lateral resolution of quantitative micro-elastography is ultimately limited by the OCT system lateral resolution (11 μm). However, the mechanical response of the sensor also impacts the ability to resolve feature boundaries; a step change in sample elasticity at the sample surface cannot result in a perfect step response in measured stress (this would require physically tearing the sensor). Rather, due to the incompressible (*i.e.*, volume-conserving) nature of the sensor material (as quantified by Poisson’s ratio ~0.5), a gradient of stress is present at feature boundaries, as seen in optical palpation^24^,^37^. This could be partially alleviated by a more compressible sensor material, such as polyacrylamide gel, which can be made to have Poisson’s ratio lower than that of silicone (~0.3 compared to ~0.5)^54^. The sample geometry also impacts on the ability to resolve features. For example, the stress detected at the surface for features deep in the sample will be diffused over a wider area than for features closer to the surface.

The dynamic range of quantitative micro-elastography depends on the strain dynamic range^41^ and the sensor elasticity. For the OCE system in this study, with minimum and maximum measurable strains of 10 με and 5 mε, respectively (see Supplementary Note), and for a sensor elasticity of 2 kPa, the theoretical minimum and maximum measurable elasticities are 4 Pa and 1 MPa, respectively (an elasticity dynamic range of ~54 dB). Changing the sensor elasticity will shift these values. Near the limits of the dynamic range, the SNR of the elasticity measurement will be low, as the strain noise floor is approached in either the sensor or the sample. Optimal elasticity SNR is achieved when the sensor and sample elasticities are matched; that is, when the strain SNR^41^ may be maximized in both sensor and sample. Thus, when designing a stress sensor for a given application, it is important to consider the expected range of sample elasticities. In addition to optimizing the SNR, a sensor with elasticity similar to or softer than that of the sample will more easily conform to an uneven sample surface, which is crucial for obtaining useful measurements. For samples exhibiting a wide range of elasticity values, such as the breast tissues measured here, it may be possible to extend the dynamic range by employing a multi-layer sensor, in which layers with varying elasticity undergo strain in response to corresponding levels of elasticity in the sample.

As observed in Figs 6 and 7, negative elasticity values can be measured, which implies that the strain in either the sensor or sample is in the direction opposite to the applied compression. We observed that this tends to occur at feature boundaries; for example, adjacent regions of opposite strain were measured at the boundaries of breast ducts using compression OCE^34^,^36^, and expansion of the stress sensor was measured adjacent to the boundary of a stiff lesion using optical palpation^24^. Such instances may be due to the incompressible nature of both tissue and the sensor material; axial compression is accompanied by lateral expansion and causes adjacent material to be pushed axially in the opposite direction. Finite element modelling of quantitative micro-elastography could elucidate the origins of this phenomenon and be used to investigate methods for its compensation. Even in cases where the assumption of uniaxial compression breaks down, the axial component of stress and strain, along with the tissue structure determined from OCT images, could be input into an inverse solution for the sample elasticity.

Although tissues exhibit nonlinear stress-strain behaviour, here elasticity was imaged at a single level of bulk preload. Note, however, that actual preload varied spatially in samples with uneven thickness. Thus, depending on the local sample nonlinearity, this non-uniformity could have resulted in over- or under-estimation of local elasticity relative to that at the bulk preload. For instance, the fibroadenoma exhibited a nodular structure with highly uneven thickness, and a spatially varying preload was necessarily imparted to make uniform contact. In future studies, we could more fully characterize the mechanical response of samples by measuring stress and strain at a number of preloads, effectively generating local “stress-strain curves” of the sample, from which nonlinear parameters could be estimated. Imaging of mechanical nonlinearity has been shown to improve tissue contrast in ultrasound elastography^55^. However, this technique would generate larger amounts of data, making rapid 3D imaging in clinical scenarios more challenging.

As only the axial component of tissue displacement is measured in quantitative micro-elastography, an implicit assumption is that the mechanical properties of tissue are isotropic. This assumption is made in the majority of elastography techniques^19^. Quantitative micro-elastography could be extended to quantify mechanical anisotropy by incorporating methods to measure all three components of displacement. This could be achieved, for example, using speckle tracking^19^.

In conclusion, we have demonstrated quantitative imaging of tissue elasticity by combining strain imaging using compression OCE with stress imaging using a compliant stress sensor. We demonstrated the ability to differentiate, based on elasticity, tissue types in human breast tumours, which, combined with the structural information afforded by OCT, provides rich diagnostic information about tissue and has the potential to contribute to improved guidance of breast-conserving surgery. Quantitative micro-elastography also extends the capabilities of compression OCE for longitudinal imaging, such as monitoring the response of disease to treatment, and has the potential to image tissue elasticity in a range of clinical applications, including the potential for *in vivo* imaging using handheld probes. Finally, beyond clinical imaging, this technique could be extended to optical coherence microscopy versions of elastography^56^ to quantify elasticity at the cellular level, opening up possibilities for *in situ* imaging of cell mechanics for biological research.

## Methods

### Spectral-domain OCT system

A custom-built, fibre-based, spectral-domain OCT system was used for all measurements. The system employs a superluminescent diode source with mean wavelength of 835 nm and 50 nm bandwidth, illuminating the sample with 10 mW of optical power. The measured axial and lateral resolutions (full-width at half-maximum irradiance) are 7.8 μm (in air) and 11 μm, respectively. The interference spectrum for each axial scan was captured with an exposure time of 2–10 μs on a CMOS line-scan camera. The system was operated in a common-path configuration^57^, with the reflected beam from the back surface of a 3-mm thick glass window used as a reference. Lateral scanning in the sample arm was performed using an *x-y* galvo-scanning mirror pair. The phase sensitivity of the system was measured to be 7.2 milli-radians at an OCT SNR of 50 dB, corresponding to a displacement sensitivity of 340 pm^34^. Displacement between scans, *d*, is related to the change in phase, 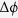, by 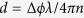
30, where *λ* is the source wavelength and *n* is the sample refractive index (assumed to be 1.4 for both silicone and tissue).

### Loading and imaging parameters

To perform imaging, the stress sensor was placed between the glass window (40-mm diameter) and the sample. Polydimethylsiloxane (PDMS) oil was applied as a lubricant at the sensor-window interface to reduce friction and ensure uniaxial compression of the sensor^45^. When the sample was a silicone phantom (Figs 1–5), PDMS oil was also used to lubricate the sensor-sample interface. When the sample was tissue, a saline solution was used to keep the tissue hydrated and also served as a lubricant. A preload was applied to ensure uniform contact as described above. Samples were then loaded quasi-statically using a piezoelectric ring actuator (Piezomechanik, Germany) to enable same-side imaging and loading. The actuator was driven at a frequency in the range 2–25 Hz. The driving frequency was chosen to remain in the quasi-static loading regime, *i.e.*, low enough to avoid wave propagation in the sample^58^.

For phantom imaging (Figs 1–5), 10 B-scans per *y*-location were acquired to enable temporal averaging of the phase signal, improving the displacement estimation accuracy, and the *x*- and *y*-locations were sampled every 5 μm and subsequently averaged to result in 10-μm *x*- and *y*-pixel size. For imaging of breast tissue, 2 B-scans per *y*-location were acquired, *x*-locations were spaced by 10 μm, and *y*-locations were spaced by 2 μm and subsequently spatially averaged to result in 10-μm *x*- and *y*-pixel size. Total acquisition time for a 10 × 10 × 2.25 mm volume (*x* × *y* × *z*) varied in the range 3–6 minutes depending on the sampling density. For future intraoperative deployment, acquisition times could be reduced to as little as 5 s per volume by taking the phase difference between unloaded and loaded C-scans instead of B-scans^23^.

Stress, strain, and elasticity images were generated using the procedure outlined in Fig. 1. For depth-resolved strain estimation, linear regression to displacement was performed over axial ranges of 100 μm (defining the axial spatial resolution of the 3D quantitative micro-elastograms). All images were scaled to physical lengths (*i.e.*, not optical path lengths) in the axial (*z*) dimension assuming a refractive index of 1.4 for all samples. To fuse the OCT and quantitative micro-elastograms for display in Figs 6 and 7, we first segmented out the adipose tissue and negative elasticity values from the quantitative micro-elastograms using image processing software (GNU Image Manipulation Program, v2.8.2). The remaining elasticity data is overlaid on the OCT image and its transparency is set to 40%.

### Phantom and stress sensor fabrication and characterization

Stress sensors and phantoms were fabricated using silicone elastomers, namely, combinations of Elastosil RT601, Elastosil P7676, and AK50 Silicone Fluid (Wacker, Germany). All phantoms were cylindrical and 15 mm in diameter. The homogeneous phantoms for the validation measurements (Fig. 3) were cured to 1 mm thickness. The bilayer phantom was made by curing a 600-μm layer of soft silicone onto a 500-μm layer of stiff silicone. The inclusion phantom was fabricated by first curing a 250-μm bottom layer of matrix material, placing the inclusion (~750 μm cubic, hand-cut from a bulk of cured silicone) on top, and curing a further layer of matrix material to cover the inclusion, resulting in a total thickness of 1.5 mm. To ensure that layers and inclusions were distinguishable in OCT images, titanium dioxide particles were added to the uncured silicones in concentrations of 1 mg/mL for the matrix material and top layer, and 2.5 mg/mL for the inclusion and bottom layer. Stress sensors were fabricated by curing a thin slab of silicone, without scatterers incorporated, to the desired thickness (between 400–600 μm). The stress sensors were 25 mm in diameter for the phantom measurements, and 50 mm in diameter for the tissue measurements.

The mechanical properties of the cured silicones were controlled by varying the volumetric ratio of crosslinker, catalyst, and silicone fluid^59^. Silicones were characterized using standard compression tests on an Instron materials testing system with 50-mm diameter platens. Silicones used for fabrication of phantoms were tested with the relevant amounts of titanium dioxide scatterers incorporated. For the stress sensor materials, stress-strain curves were generated for three different batches of each material to test for inter-batch variability, and at least three curves were generated for each batch to test for inter-measurement variability. We found the elasticity to vary by less than 10% in the strain range 0–0.2 (see Supplementary Figure S2 as an example). For the validation measurements (Fig. 3), in order to maximize the elasticity SNR, we closely matched the sensor and phantom elasticity in each case by using the same silicone material for both sensor (no scatterers incorporated) and phantom (2 mg/mL scatterers incorporated). For heterogeneous phantom measurements, we used a sensor material with elasticity intermediate between the stiff and soft features. For tissue measurements, in order to ensure that the sensor would conform to the soft tissue surface, we used a soft sensor material with elasticity <10 kPa at strains <0.5. This elasticity is similar to that of breast adipose tissue^40^,^44^.

### Preparation of breast tissue samples

Informed consent was obtained from patients and the study approved by the Human Research Ethics Committee of Royal Perth Hospital, Perth, Western Australia. Experimental procedures were performed in accordance with approved guidelines. Fresh, unstained tissues were obtained from patients undergoing surgical removal of malignant tumour (invasive ductal carcinoma) or fibroadenoma. A pathologist (BL) extracted samples of ~2 × 2 × 1 cm in size from the excised tumour masses, and measurements were performed within three hours of excision. Prior to and throughout imaging, samples were kept hydrated using saline. Following measurements, H&E stained sections of the tissue were prepared using standard histological techniques.

## Additional Information

**How to cite this article**: Kennedy, K. M. *et al.* Quantitative micro-elastography: imaging of tissue elasticity using compression optical coherence elastography. *Sci. Rep.* **5**, 15538; doi: 10.1038/srep15538 (2015).

## Supplementary Material

Supplementary Information
