# Supplementary material for: Quantitative micro-elastography: imaging of tissue elasticity using compression optical coherence elastography
Source: Sci Rep. 2015 Oct 27;5:15538. doi: 10.1038/srep15538 (PMC4622092; doi:10.1038/srep15538)
Supplement: Supplementary Information [file srep15538-s1.pdf]

Quantitative micro-elastography: imaging of tissue elasticity using compression optical coherence elastography

Kelsey M. Kennedy, Lixin Chin, Robert A. McLaughlin, Bruce Latham, Christobel M. Saunders, David D. Sampson, and Brendan F. Kennedy

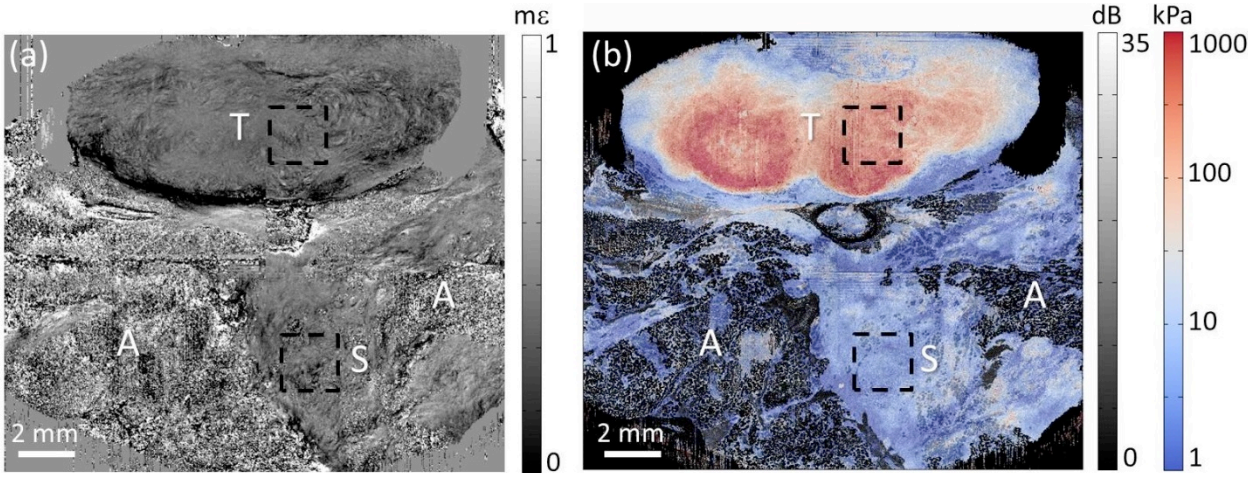

Supplementary Figure S1. Demonstration of improved mechanical contrast in quantitative micro-elastography compared to qualitative compression OCE. In (a), the strain image generated for the breast result shown in Fig. 6 of the main manuscript is presented. In (b), the corresponding elasticity image presented in Fig. 6(c) is reproduced to facilitate comparison with the strain image in (a). In this example, there is much higher contrast in the elasticity image than in the strain image. This additional contrast was quantified by calculating the mean strain and elasticity in the black boxes and calculating the ratio in both cases. In the strain image, this ratio is 0.77 and in the elasticity image it is 82, corresponding to an increased contrast of >100 in the elasticity image.

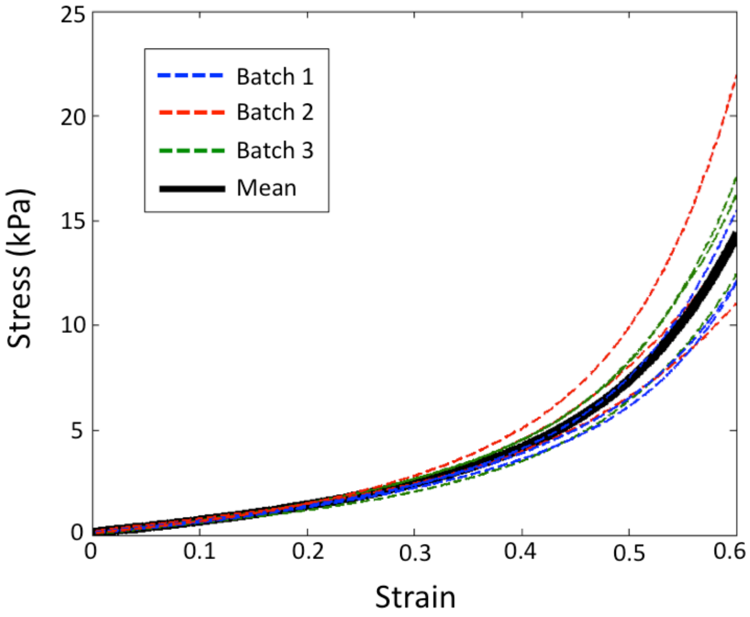

Supplementary Figure S2. Stress-strain curve repeatability for silicone sensor material. Measurements were obtained on an Instron materials testing machine using a load cell calibrated to an accuracy of 0.01 N over a range 1-100 N, for nine samples of the most commonly used sensor material, Elastosil P7676 in a catalyst:crosslinker:fluid ratio of 2:1:0.3. Curves are shown for three samples from each of three separate batches, indicated by the blue, red, and green dashed lines. The mean curve, indicated by the black solid line, was used to estimate stress in the quantitative micro-elastography experiments. As a measure of repeatability, the slope of a linear fit to each stress-strain curve over the range 0-0.2 strain results in a mean of 1.5 kPa and a standard deviation of 0.1 kPa (<7%).

Supplementary Note 1

The sensitivity and dynamic range of quantitative micro-elastography depends on the strain sensitivity and strain dynamic range of the OCE system[1] and on the relative mechanical properties of the sensor and sample. For a mechanically homogeneous sample,

assuming the sensor and sample behave as a system of springs (*i.e.*, strain is inversely proportional to elasticity), Equation 2 in the manuscript can be expanded as:

$$E_{sample} = \frac{\sigma_{sensor}}{\varepsilon_{sample}} = \frac{E'_{sensor}\varepsilon_{sensor}}{\varepsilon_{sample}} = \frac{E'_{sensor}d_l}{l\varepsilon_{sample}}, \tag{S1}$$

where E'_{sensor} is the slope of the tangent to the stress-strain curve of the sensor material at the point of preload, d_l is the displacement at the sensor-sample interface, and l is the sensor thickness. Considering Equation S1, upper and lower limits to measurable elasticity can be defined for a given sensor elasticity and given OCE system parameters. The maximum measurable elasticity is that corresponding to the highest measurable strain (and, therefore, stress) in the sensor and the lowest measurable strain in the sample, and vice versa for the lowest measurable elasticity. For our system, the highest measurable strain using phase-sensitive detection of displacement is 5 mε. Above this strain magnitude, the speckle patterns in consecutive pairs of B-scans used to determine displacement begin to decorrelate, degrading the strain SNR[2]. The strain sensitivity using our system and acquisition parameters is 10 με, calculated as the standard deviation of the strain over 500 μm in a homogeneous phantom at OCT SNR 20 dB (see Chin *et al.*[2] for a further discussion of experimental and processing parameters impacting strain dynamic range). Any strain value below this is considered to be below the noise floor of our measurement, and will not yield an accurate estimate of elasticity. For a sensor elasticity of 2 kPa (this value is typical of the stress sensor materials used in this study, at ~0.1 strain due to preload), this sets a theoretically measurable elasticity range of 4 Pa to 1 MPa.

Equation S1 also reveals that elasticity sensitivity is inversely proportional to sensor thickness; that is, a thicker stress sensor will allow detection of smaller values of elasticity.

However, the requirement to image through the sensor into the sample sets a practical limit on the maximum thickness of the sensor. Here, we have used sensors with thicknesses less than the Rayleigh range of the OCT system in silicone, 640 μm, in order to obtain high quality OCT images of the underlying sample, as well as ensure accurate strain estimation.

[1] Kennedy, B. F. *et al.* Strain estimation in phase-sensitive optical coherence elastography. *Biomedical Optics Express* **3**, 1865-1879 (2012).

[2] Chin, L. *et al.* Analysis of image formation in optical coherence elastography using a multiphysics approach. *Biomedical Optics Express* **5**, 2913-2930 (2014).
